# The Structure of the Karrikin-Insensitive Protein (KAI2) in *Arabidopsis thaliana*

**DOI:** 10.1371/journal.pone.0054758

**Published:** 2013-01-18

**Authors:** Rohan Bythell-Douglas, Mark T. Waters, Adrian Scaffidi, Gavin R. Flematti, Steven M. Smith, Charles S. Bond

**Affiliations:** 1 School of Chemistry and Biochemistry, The University of Western Australia, Crawley, WA, Australia; 2 Australian Research Council Centre of Excellence for Plant Energy Biology, The University of Western Australia, Crawley, WA, Australia; Plant and Food Research, New Zealand

## Abstract

KARRIKIN INSENSITIVE 2 (KAI2) is an α/β hydrolase involved in seed germination and seedling development. It is essential for plant responses to karrikins, a class of butenolide compounds derived from burnt plant material that are structurally similar to strigolactone plant hormones. The mechanistic basis for the function of KAI2 in plant development remains unclear. We have determined the crystal structure of *Arabidopsis thaliana* KAI2 in space groups *P*2_1_ 2_1_ 2_1_ (a  = 63.57 Å, b  = 66.26 Å, c  = 78.25 Å) and *P*2_1_ (a  = 50.20 Å, b  = 56.04 Å, c  = 52.43 Å, β  = 116.12°) to 1.55 and 2.11 Å respectively. The catalytic residues are positioned within a large hydrophobic pocket similar to that of DAD2, a protein required for strigolactone response in *Petunia hybrida*. KAI2 possesses a second solvent-accessible pocket, adjacent to the active site cavity, which offers the possibility of allosteric regulation. The structure of KAI2 is consistent with its designation as a serine hydrolase, as well as previous data implicating the protein in karrikin and strigolactone signalling.

## Introduction

Bushfires present organisms with both hardships and opportunities. For plants, fires deliver a flux of nutrients to the soil and reduce competition for light and water. Karrikins are a class of compound present in bushfire smoke that stimulate the germination of dormant seeds [1], [2]. Karrikins are characterised by a butenolide moiety that is also common to strigolactones, endogenous plant hormones that were originally identified as factors that promote germination of seeds of parasitic weeds of the Orabanchaceae such as *Striga* and *Orobanche* species [3], [4]. Strigolactones stimulate hyphal branching of arbuscular mycorrhizal fungi [5]–[7], promoting a symbiotic interaction with more than 80% of vascular plants that enhances nutrient uptake. In addition, strigolactones regulate several aspects of shoot and root development and thus influence overall plant architecture [8]–[12].

Discovering the molecular mechanisms of karrikin and strigolactone action has recently taken a step forward with the isolation of plant mutants. A family of α/β hydrolases has emerged as central players in mediating the responses of plants to these butenolide compounds. The *dwarf14* mutant of rice is insensitive to strigolactones, as are orthologous mutants in Arabidopsis and petunia [13]–[15]. DAD2, the D14 orthologue in petunia, has slow hydrolytic activity towards the synthetic strigolactone GR24 (∼3 molecules of GR24 hydrolysed per DAD2 molecule per hour [15]) and this activity has been proposed to be essential for strigolactone perception [15]. Furthermore, the direct hydrolysis of the butenolide ring has recently been proposed as the mode of action for strigolactones and extended to karrikins [16]. In Arabidopsis, the DWARF14 paralogue KAI2 (KARRIKIN INSENSITIVE 2) is required for responses to karrikins, but does not appear to be essential for strigolactone responses, at least with respect to the regulation of shoot branching [14]. Nevertheless, genetic studies indicate that KAI2 can mediate activity of compounds besides karrikins, including GR24 and other butenolides with strigolactone-like functionality [14], [17]. Structural specialisation between DWARF14/DAD2 and KAI2 proteins may explain their functional specificities towards different butenolides.

The precise role that KAI2 plays in the karrikin and strigolactone signalling pathways remains unclear. Here we report the crystal structure of KAI2, which may provide valuable insight into its involvement in these pathways, especially with regard to substrate or ligand specificity. In particular, a comparison with the recently elucidated structure of DAD2 and its postulated role as both a strigolactone receptor and hydrolase is discussed.

## Materials and Methods

### Synthesis of KAR_2_

KAR_2_ was prepared according to the method of Goddard-Borger *et al*. [18].

### Cloning and expression of KAI2

The native *Arabidopsis thaliana* KAI2 coding sequence (At4g37470) was amplified by PCR using seedling-derived cDNA template and primers 5′–GGGGACAAGTTTGTACAAAAAAGCAGGCTTC**ATG**GGTGTGGTAGAAGAAGC–3′ and 5′–GGGGACCACTTTGTACAAGAAAGCTGGGTT**TCA**CATAGCAATGTCATTAC–3′ (start and stop codons, respectively, are highlighted in bold; Gateway recombination sites are underlined), and subsequently cloned into the pDEST17 expression vector (Life Technologies). The expression clone was introduced into the Rosetta strain (Novagen, Darmstadt). Cultures (400 ml) were grown in SOC medium at 37°C to OD_600_ ∼0.6. At this point, the cultures were cooled to 16°C and recombinant protein expression was induced by the addition of 0.1 mM IPTG. Growth proceeded for a further 18 hours at 16°C before harvesting by centrifugation. Wet pellets were frozen in dry ice and stored at −80°C until processing.

### Preliminary Purification

Cell pellets were resuspended in 50 mM sodium phosphate, 500 mM NaCl pH 8.0, at a ratio of 5 mL lysis buffer to 1 g of cell pellet and supplemented with 1 unit Benzonase nuclease (Novagen, Darmstadt) per mL lysis buffer. Cells were lysed with detergents (1x BugBuster® (Novagen, Darmstadt) at room temperature (20°C) with shaking at ∼60 rpm for 20 minutes. The lysate was clarified by centrifugation at 16,000×g for 20 minutes at 4°C. To isolate His-tagged protein the clarified lysate was combined with 3 mL 50% (v/v) slurry of pre-equilibrated nickel-nitrilotriacetic acid resin (Qiagen) which was mixed end-over-end for 80 min at 4°C and 20 rpm. The bound KAI2 was washed twice with 10 mL 50 mM sodium phosphate, 500 mM NaCl, 20 mM imidazole pH 8.0 by gravity flow. KAI2 protein was eluted with 5 mL 50 mM sodium phosphate, 500 mM NaCl, 250 mM imidazole pH 8.0. Eluted protein was dialysed against 2 L 20 mM sodium phosphate 150 mM NaCl pH 8.0. Dialysed KAI2 protein was recovered at 950 μg/mL and used for optimisation of solubility. Protein concentrations were determined by spectrophotometric measurements at 280 nm using a calculated molar extinction coefficient (http://web.expasy.org/protparam/) [19] on samples diluted ten-fold in 8 M urea.

### Optimisation of protein solubility

The instability of KAI2 at concentrations greater than 1 mg/mL in 20 mM sodium phosphate buffer, 150 mM NaCl pH 8.0, prompted optimum solubility screening. The buffer solutions screened against were citric acid pH 3.0–6.0, sodium acetate pH 4.0–5.5, MES pH 5.3, Bis-Tris pH 5.0–7.0, MOPS pH 6.5–7.4, HEPES pH 6.5–8.0 or Tris pH 6.5–9.0, at 0.1 M concentration with either 75 or 150 mM NaCl. Screening for the buffer with optimal protein solubility was performed according to Jancarik *et al* [20], which is the same format as a hanging drop vapour diffusion crystallisation experiment. Briefly, drops containing 5 μL of protein solution were mixed with 5 μL of buffer solution and equilibrated against 500 μL of buffer solution at 293 K. After 4 weeks, the drops containing 0.1 M Tris pH 8.5 and 9.0 supplemented with 75 and 150 mM NaCl had remained clear. These buffers were used in subsequent purifications of KAI2.

### KAI2 purification

Pellets from 400 mL of expression culture were resuspended with 15 mL of 100 mM Tris 300 mM NaCl 20 mM imidazole pH 9.0, supplemented with 15 units of Benzonase (Novagen, Darmstadt). Cells were lysed with 4 passes through an Emulsiflex C5 high-pressure homogeniser at 10,000–15,000 kPa (Avestin, Ottawa). Lysates were clarified by centrifugation at 16,100 rcf for 1 h at 4°C. All chromatography steps were performed at room temperature. The clarified lysate was filtered through a 0.22 μm filter and applied manually to a pre-equilibrated 5 mL HisTrap column (GE Healthcare). The loaded column was washed with four column volumes of lysis buffer at 2 mL/min. Protein was eluted using a ten column volume linear gradient from 0–100% (buffer +250 mM imidazole). KAI2 eluted at approximately 100 mM imidazole. KAI2-containing fractions were pooled, concentrated to 3.5 mL and applied to a 120 mL Superdex 200 column (GE Healthcare) for size exclusion chromatography in 100 mM Tris 150 mM NaCl pH 9.0. Pure KAI2 typically eluted as one peak, which was collected and concentrated using a 10 kDa molecular weight cut off centrifugal ultrafilter (Sartorius) to 10.2 mg/mL, yielding approximately 16 mg of highly purified KAI2 protein. In an attempt to obtain protein bound to phenylmethyl sulfonyl fluoride (PMSF), KAI2 was lysed in 20 mM Tris pH 7.0, 150 mM NaCl, 1 mM PMSF. The lysate was gently rocked at 4°C for 1 hour before 5 mL of 200 mM TRIS pH 9.0 was added and the purification was continued as previously outlined. Purified protein was further incubated with 1 mM PMSF at 4°C for 16 hours. All protein samples were divided into aliquots, flash-cooled in liquid nitrogen and stored at −80°C.

### Protein crystallisation

**Figure 1:**
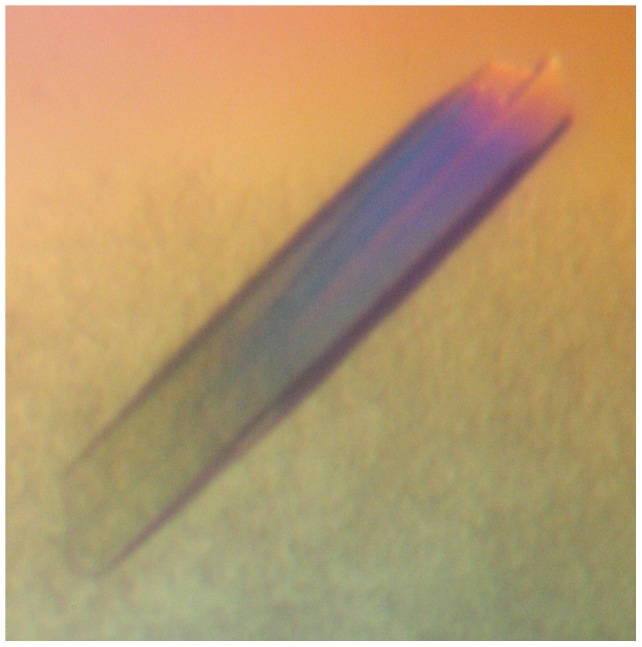
KAI2 crystal. A multiple KAI2 crystal (32 μm×6 μm×6 μm) that was split for data collection (KAI2b).

Sparse matrix vapour diffusion crystallisation screens were performed using Hampton Index Screen (Hampton Research) with a Phenix liquid handling robot (Art Robbins) in sitting drop format in 96 well plates (Hampton Research, HR8-149). Drops between 0.4 and 0.6 μL were equilibrated against 90 μL of reservoir solution. A harvestable, diffracting protein crystal grew in a condition with 0.2 μL of protein solution (10.2 mg/mL KAI2) and 0.4 μL of reservoir solution (1.4 M sodium/potassium phosphate pH 8.4). Crystals were optimised by screening buffer pH and concentration in 24 well sitting drop format where 2.5 μL of protein solution (5.0 mg/mL) was added to 2.5 μL reservoir solution and equilibrated against 500 μL of reservoir solution. A 32 μm×6 μm×6 μm multiple crystal grew in 1.5 M sodium/potassium phosphate pH 7.2 (KAI2b; Figure 1) and single crystals grew in 1.4 M sodium/potassium phosphate pH 7.3. (KAI2c). KAI2 seed stock was prepared from a crystal in the same drop that yielded KAI2b. Attempts to co-crystallise KAI2 with the karrikin molecule KAR_2_ were unsuccessful, but yielded higher resolution diffracting crystals (KAI2a). Crystals that grew in the presence of KAR_2_ were crystallised from a drop containing 1.8 μL protein sample (6 mg/mL), 0.6 μL KAI2 seed stock, 0.4 μL 30% glycerol and 1.2 μL reservoir solution of 1.4 M sodium/potassium phosphate pH 7.3. Prior to crystallisation, KAR_2_ dissolved in 100% (v/v) DMSO was added to the protein solution to a final concentration of 1 mM KAR_2_ and 2.5% DMSO. The N-terminal leader sequence was not removed prior to crystallisation experiments.

### Data collection and processing

Where necessary, single crystals were split from the multiple crystals. Crystals mounted in a nylon loop were briefly immersed in mother liquor containing 20% glycerol then frozen in liquid nitrogen for data collection. Complete X-ray data (KAI2a –180° in 0.5° rotations, KAI2b and KAI2c –360° in 1.0° rotations) were collected at the Australian Synchrotron beamlines MX1 or MX2.

Data were integrated with XDS [21] and scaled using SCALA [22] from the CCP4 software suite [23]. The structure was solved by molecular replacement with MOLREP [24] using the crystal structure of the monomeric *Bacillus subtilis* protein RsbQ (PDB code 1WOM) [25] as the search model. Model building was performed with COOT [26]. Initial rigid-body and restrained refinement was performed using REFMAC [27]. Final rounds of refinement were performed with BUSTER [28]. Root-mean-square deviation (RMSD) values were calculated with LSQMAN [29]. Cavity volumes were calculated using VOIDOO [30] on the highest resolution structure KAI2a, using a primary grid spacing of 0.2 Å. Molecular graphics were generated using PYMOL [31]. Structures were analysed using MOLPROBITY [32]. Atomic coordinates and structure factors have been deposited in the Protein Data Bank under accession codes 4HRY (KAI2a), 4HTA (KAI2b) and 4HRX (KAI2c).

### Protein sequence analysis

Protein sequence alignments were performed using ALINE [33]. KAI2 protein sequences used in sequence alignments were from *Arabidopsis thaliana* (NCBI GI: 15235567), *Ricininus communis* (NCBI GI: 255567989), *Populus trichocarpa* (NCBI GI: 224071259), *Solanum lycopersicum* (NCBI GI: 225311281), *Vitis vinifera* (NCBI GI: 225458830), *Brachypodium distachyon* (PlantGDB: Brachypodium_distachyon-10841), *Hordeum vulgare* (NCBI GI: 326500818), *Zea mays* (NCBI GI: 226530032), *Sorghum bicolor* (NCBI GI: 242035387) and *Oryza sativa* cv. *japonica* (NCBI GI: 115453689). D14 protein sequences used in sequence alignments were from *Petunia hybrida* (DAD2) (NCBI GI: 404434487), *Arabidopsis thaliana* (NCBI GI: 18396732), *Ricinus communis* (NCBI GI: 255538072), *Vitis vinifera* (NCBI GI: 225458830), *Populus trichocarpa* (NCBI GI: 224129864) *Solanum lycopersicum* (Sol genomics network: Solyc04g077860.2.1), *Hordeum vulgare* (NCBI GI: 326496392), *Oryza sativa* cv. *japonica* (NCBI GI: 115451411), *Sorghum bicolor* (NCBI GI: 242041843) and *Zea mays* (NCBI GI: 226501208).

## Results and Discussion

**Figure 2:**
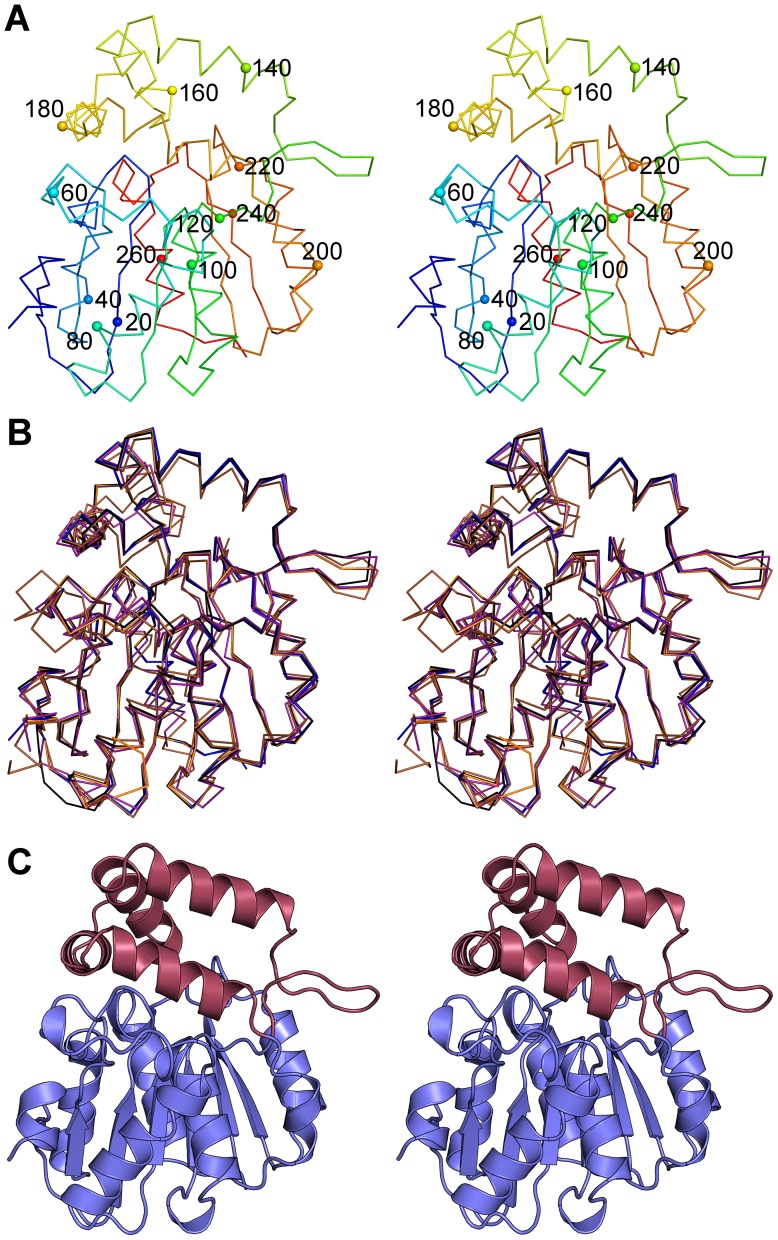
Crystal structure of KAI2. A. Stereoscopic ribbon diagram of KAI2 coloured from amino (blue) to carboxy (red) terminus. Every twentieth Cα is shown as a labelled sphere. B. Stereoscopic ribbon diagram of all three models of KAI2 (KAI2a blue, KAI2b orange, KAI2c black) and the models of DAD2 (purple) and RsbQ (brown) superposed. C. Stereoscopic cartoon diagram of KAI2. The α/β hydrolase domain is shown in blue and the cap domain shown in red.

*A. thaliana* KAI2 was cloned into pDEST17 and expressed in *E. coli* resulting in protein samples of approximately 30 kDa molecular mass as determined by SDS-PAGE. An optimal buffer for solubility was identified as 0.1 M Tris pH 9.0, 150 mM NaCl. KAI2 was successfully crystallised from sodium/potassium phosphate in two crystal forms, one orthorhombic, *P* 2_1_ 2_1_ 2_1_ (a∼64 Å, b∼66 Å, c∼78 Å) and the other monoclinic, *P* 2_1_ (a∼50 Å, b∼56 Å, c∼52 Å, β∼116°), each with one protein molecule in the asymmetric unit. Full details of data collected from three crystals (KAI2a, KAI2b and KAI2c) are presented in Table 1. The processed KAI2a data showed a high signal-to-noise ratio at high resolution indicating that the crystal diffracted beyond the collected limit of 1.55 Å. Unfortunately, the crystal had deteriorated before higher-resolution data could be collected, and other crystals did not diffract so well. KAI2a is presented here as the highest resolution structure (1.55 Å)(Figure 2A). Although KAI2b diffracted less well (1.90 Å), a portion of the N-terminal linker sequence (from Ser −8 to Lys −5) could be clearly observed in the electron density. Monoclinic KAI2c diffracted to 2.11 Å.

**Table 1:** Data collection and refinement statistics for *Arabidopsis thaliana* KAI2.

Structure	KAI2.a	KAI2.b	KAI2.c
Space group	P2_1_2_1_2_1_	P2_1_2_1_2_1_	P2_1_
Unit-cell parameters (Å, °)	a = 63.57, b = 66.26, c = 78.25,	a = 63.39, b = 66.06, c = 77.62,	a = 50.20, b = 56.04, c = 52.43, β = 116.12
Temperature (K)	100	100	100
X-ray source	MX1, Australian Synchrotron	MX2, Australian Synchrotron	MX2, Australian Synchrotron
X-ray wavelength (Å)	0.95370	0.95390	0.95390
Detector	ADSC Quantum 210r CCD	ADSC Quantum 315r CCD	ADSC Quantum 315r CCD
Resolution (Å)	27.50–1.55 (1.64–1.55)	77.62–1.90 (2.00–1.90)	56.04–2.11 (2.23–2.11)
R_merge_ (%)	9.3 (54.2)	11.5 (68.0)	16.2 (65.9)
R_meas_ (%)	10.2 (59.1)	12.3 (70.6)	17.4 (71.1)
R_pim_ (%)	4.0 (23.1)	3.2 (18.6)	6.3 (26.4)
No. of unique reflections	48612 (6723)	26147 (3575)	14659 (1896)
Average multiplicity	6.5 (4.0)	14.3 (13.9)	7.4 (6.7)
(*I/σ(I))*	10.8 (3.0)	22.5 (4.9)	11.1 (2.8)
Refinement	-	-	-
* Rwork*	18.10	15.52	16.09
* Rfree*	20.65	17.40	20.63
Mean *B* value (Å^2^)	20.04	22.48	22.19
R.m.s.d. from ideal geometry	-	-	-
Bond lengths (Å)	0.010	0.010	0.010
Bond angles (°)	1.010	0.96	1.070
No. of protein residues	268	271	266
Water/solvent atoms	405	272	194
Estimated coordinate error (Luzzati) (Å)	0.164	0.159	0.205
Poor rotamers^a^	0	0	0
Ramachandran^a^	-	-	-
Favoured (%)^a^	97.7%	98.5%	98.1%
Allowed (%)^a^	2.3%	1.5%	1.9%

Values in parentheses correspond to the highest resolution shell. ^a^ Values obtained using MOLPROBITY [32].

No conspicuous conformational differences were observed between KAI2 structures (Figure 2B; maximum RMSD between KAI2a and KAI2c, 0.4 Å [265/269 Cα atoms]). The overall fold of KAI2 consists of an α/β hydrolase domain and a four helix cap domain (Figure 2C). KAI2 has essentially identical topology to DAD2 [15] and the signalling protein RsbQ from *Bacillus subtilis* [25] (all missing the first β-strand of the canonical α/β hydrolase domain [34]), with RMSD values of 0.9 Å [262/269 Cα atoms] and 1.2 Å [254/269 Cα atoms] from KAI2a respectively.

### The protein surface

Functional specialisation between KAI2 and D14 proteins might be mediated by each of the proteins' respective interaction partners, so we scrutinised the conserved amino acid differences of surface exposed residues within each of the protein families. No conspicuous regions of conserved differences were detected which would indicate sites of interaction with different protein partners.

### The active site cavity

**Figure 3:**
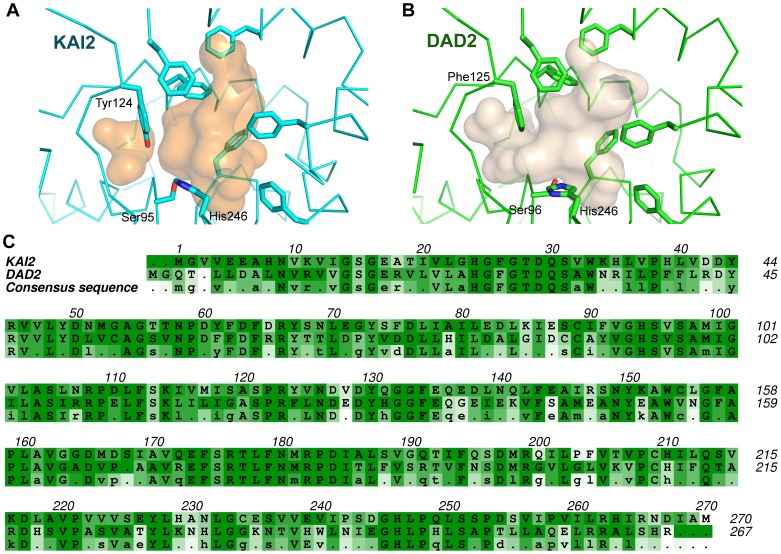
Comparing KAI2 with DAD2. The active site cavities of KAI2 (A) and of DAD2 (B). Cavity-exposed phenylalanine and tyrosine residues are shown for both proteins. Tyr124 of KAI2 occludes a small adjacent pocket that is not occluded in DAD2. Other than this difference, the two pockets are similar in size and shape. C. Sequence alignments of KAI2 and DAD2. The *A. thaliana* KAI2 sequence is coloured by similarity within the KAI2 protein family, the *P. hybrida* DAD2 sequence is coloured by similarity within the D14 protein family and the consensus sequence was determined and coloured by similarity across both families. Darker green colouration indicates more conserved sequence. Capital letters in the consensus sequence denote a conserved residue across all KAI2 and D14 proteins. Amino acids of interest are those where aligned KAI2 and D14 residues are coloured darker than the corresponding consensus sequence residue.

The active site is present in a largely hydrophobic pocket of volume 336 Å^3^, with a classical serine hydrolase catalytic triad at its base (composed of Ser95-His246-Asp217). The KAI2 catalytic pocket is smaller than the catalytic pocket of DAD2 (448 Å^3^), but it is still sufficiently large to accommodate the synthetic strigolactone GR24. The two pockets are highly similar in terms of overall shape and amino acid composition (Figures 3A and 3B). No obvious active site cavity features account for a difference between KAI2 and D14 in terms of karrikin binding. Six of the seven cavity-lining phenylalanine residues are conserved between the two proteins (Figures 3A and 3B). The non-conserved residue within these is Tyr124, which replaces Phe125. The hydroxyl group of this side chain occludes a small pocket (41 Å^3^) proximal to the catalytic residues. In DAD2, this small pocket is connected to the main cavity, helping to explain the discrepancy in size between the KAI2 and DAD2 main cavity volumes. This specific Tyr/Phe substitution is conserved within KAI2 and D14 protein families respectively (Figure 3C) suggesting that the two proteins may differ in their natural substrates/ligands.

### Catalytic residues

**Figure 4:**
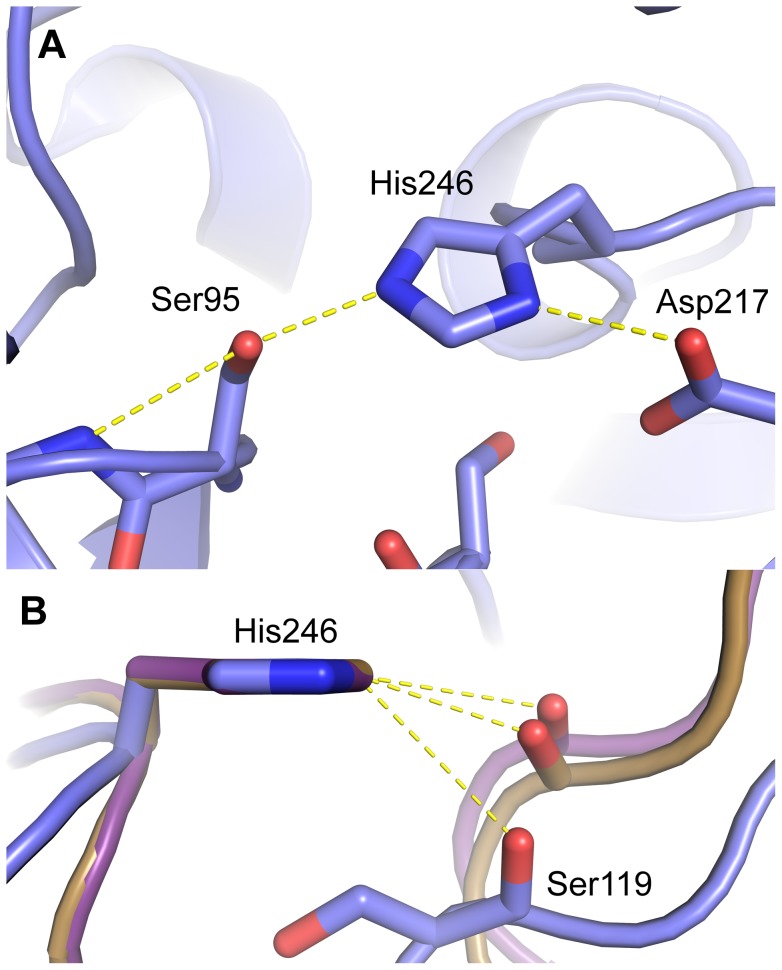
The KAI2 catalytic triad. A. The catalytic residues Ser95-His246-Asp217 are hydrogen bonded in a classical arrangement for a serine hydrolase (Residues are shown in stick representation, coloured by atom type). B. CH…O hydrogen bonding between histidine Cε1 and carbonyl oxygens. KAI2 (blue), DAD2 (purple) and RsbQ (brown) all superimposed using the imidazole ring of the catalytic histidine of each respective protein. KAI2, unlike DAD2 and RsbQ, has unfavourable hydrogen bonding geometry between the catalytic His Cε1 and the carbonyl oxygen. This hydrogen bond is important for serine hydrolase activity [37], [38].

The catalytic triad of KAI2 is observed with either a Tris buffer molecule (KAI2a) or glycerol molecule (KAI2b) nearby. As the structure of a PMSF-adduct of RsbQ had previously been reported [25], we attempted extensive incubation of KAI2 with PMSF, but were unable to detect any covalent modification at Ser95 either in crystals or by electrospray mass-spectrometry. This observation is not unprecedented for catalytically active serine hydrolases [35], [36]. In an attempt to rationalise the inactivity of KAI2 towards PMSF, we scrutinised the conformation of the catalytic residues. The Ser-His-Asp triad residues are hydrogen bonded in a classical conformation for active hydrolysis (Figure 4A) [34]. However, the imidazole side-chain of His246 is in a different plane to that observed in DAD2 and RsbQ, coordinating to Oδ1 of Asp217 as opposed to Oδ2. Furthermore, a discrepancy in the coordination of Cε1 of His246 in KAI2 compared to that of active serine hydrolases, including DAD2 and RsbQ was observed. (Cε1 of the catalytic histidine in serine hydrolases is acidic and acts as a CH…O hydrogen bond donor to a carbonyl oxygen [37] in catalytically active serine hydrolases [37], [38]). Consistent with this, Cε1 of His246 in DAD2 forms a 3.34 Å hydrogen bond to the amide O of Gly120 which deviates 9° from the Cε-H bond. In RsbQ, Cε1 of His247 forms a 3.04 Å hydrogen bond to the amide O of Gly120 which deviates 22° from the Cε1-H bond, while Cε1 of His246 in KAI2 is 3.65 Å from the amide O of Ser119 and deviates 53° from the Cε1-H bond (Figure 4B). Steric hindrance from the side chain of Ser119 in KAI2 (as opposed to Gly119 in DAD2 and Gly120 in RsbQ) alters the arrangement of the catalytic histidine, potentially explaining the inability of the protein to react with PMSF. This amino acid substitution between KAI2 and DAD2 at position 119 is conserved within KAI2 and D14 protein families (Figure 3C). The structure of DAD2 is described as having an inactive conformation at the active site (Oγ of Ser96 is oriented away from His246) [15], yet retains hydrolytic activity on GR24 [15]. Therefore some rearrangement must occur within the catalytic residues of DAD2 prior to catalysis. Similarly, the catalytic residues of KAI2 are observed in an inactive conformation and may require perturbation for activity. This may be facilitated by the natural substrate for the enzyme or some other regulatory mechanism, which cannot be mimicked by PMSF.

### A secondary pocket

**Figure 5:**
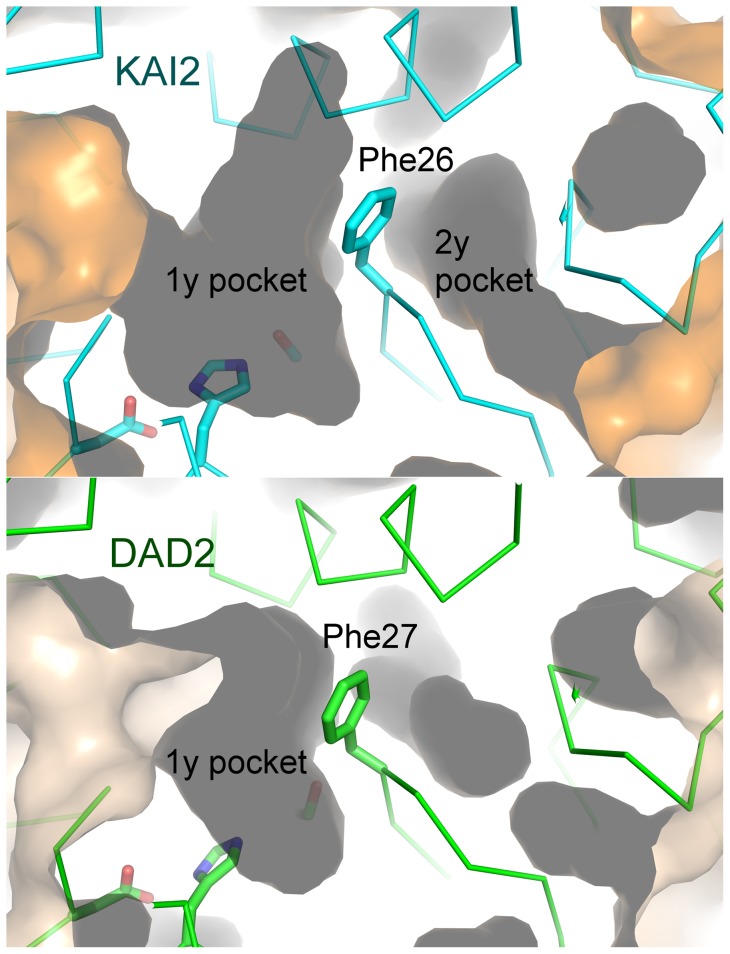
The second pocket of KAI2. A. The two pockets of KAI2a are separated internally by the aromatic side-chain of Phe26. The active site residues can be seen in the primary pocket (left). Both pockets are solvent accessible. B. The active site cavity of DAD2 and the adjacent small, non-solvent accessible pocket.

There is a second solvent-accessible pocket within KAI2, adjacent to the primary pocket containing the active site residues but separated from it internally by the side-chain of Phe26 (Figure 5A). The secondary pocket (137 Å^3^) is significantly smaller than the primary pocket but is large enough to accommodate a karrikin-sized molecule. The same pocket is present within the DAD2 structure (Figure 5B), although it is smaller (30 Å^3^) and blocked from the solvent by the side chain of Phe188. The equivalent residue in KAI2 is Ser188, which permits solvent accessibility of the pocket. This Ser/Phe substitution is conserved within KAI2 and D14 protein families (Figure 3B). The difference in size between the two pockets is accounted for by another conserved difference between the proteins at residue Gly53 of KAI2/ Cys54 of DAD2, where in DAD2 the cysteine side chain protrudes in to the second pocket, reducing its volume. The solvent accessibility, volume, and close proximity of this secondary pocket to the active site pocket offer the possibility that KAI2 activity may be regulated by an allosteric co-factor. The conserved differences between KAI2 and D14 protein families suggest that this second pocket may play a role in the functional specialisation of these two proteins.

## Conclusion

The X-ray crystal structure of KAI2 has provided us with insight into the possible molecular function of the enzyme. The conformation of active site residues supports the designation of the enzyme as a serine hydrolase, although the conditions under which it is active and its native substrate remain unknown. The KAI2 active site cavity is large enough to accommodate a strigolactone molecule and is highly similar in structure and amino acid composition to the strigolactone hydrolase DAD2. This observation is consistent with genetic studies that indicate that KAI2 and AtDWARF14 (the Arabidopsis DAD2 orthologue) can both mediate seedling responses to the synthetic strigolactone GR24 [14]. However, AtDWARF14 is unable to mediate karrikin signalling, and karrikins cannot function as inhibitors of shoot branching, unlike GR24 [39]. There are no conspicuous features within the KAI2 structure that explain the difference in karrikin perception between KAI2 and D14 proteins. An inspection of the conserved differences in surface amino acid composition between KAI2 and D14 yielded no conspicuous regions of divergence. The unexpected finding of a second solvent-accessible pocket, distinct from, yet in close proximity to the active site pocket suggests that the activity of KAI2 might be regulated by an allosteric cofactor. This second pocket is larger than the equivalent pocket in DAD2 which is not solvent accessible, suggesting that this second pocket may contribute to functional specialisation between KAI2 and D14 proteins. The elucidation of the KAI2 structure now opens up avenues for investigating this possibility.

## References

[1] Flematti GR, Ghisalberti EL, Dixon KW, Trengove RD (2004) A compound from smoke that promotes seed germination. Science 305: 977. 15247439 10.1126/science.1099944

[2] Nelson DC, Flematti GR, Ghisalberti EL, Dixon KW, Smith SM (2012) Regulation of seed germination and seedling growth by chemical signals from burning vegetation. Annu Rev Plant Biol 63: 107–130. 22404467 10.1146/annurev-arplant-042811-105545

[3] Cook CE, Whichard LP, Turner B, Wall ME, Egley GH (1966) Germination of Witchweed (*Striga lutea* Lour.): Isolation and properties of a potent stimulant. Science 154: 1189–1190. 17780042 10.1126/science.154.3753.1189

[4] Cook CE, Whichard LP, Wall ME, Egley GH, Coggon P, et al (1972) Germination Stimulants.II. Structure of strigol a potent seed-germination stimulant for witchweed (*Striga-Lutea* Lour). J Am Chem Soc 94: 6198–6199.

[5] Besserer A, Puech-Pagès V, Kiefer P, Gomez-Roldan V, Jauneau A, et al (2006) Strigolactones stimulate arbuscular mycorrhizal fungi by activating mitochondria. PLoS Biol 4: e226. 16787107 10.1371/journal.pbio.0040226 PMC1481526

[6] Yoshida S, Kameoka H, Tempo M, Akiyama K, Umehara M, et al (2012) The D3 F-box protein is a key component in host strigolactone responses essential for arbuscular mycorrhizal symbiosis. New Phytol 196: 1208–1216. 23025475 10.1111/j.1469-8137.2012.04339.x

[7] Akiyama K, Matsuzaki K, Hayashi H (2005) Plant sesquiterpenes induce hyphal branching in arbuscular mycorrhizal fungi. Nature 435: 824–827. 15944706 10.1038/nature03608

[8] Gomez-Roldan V, Fermas S, Brewer PB, Puech-Pages V, Dun EA, et al (2008) Strigolactone inhibition of shoot branching. Nature 455: 189–194. 18690209 10.1038/nature07271

[9] Kapulnik Y, Delaux PM, Resnick N, Mayzlish-Gati E, Wininger S, et al (2011) Strigolactones affect lateral root formation and root-hair elongation in *Arabidopsis* . Planta 233: 209–216. 21080198 10.1007/s00425-010-1310-y

[10] Umehara M, Hanada A, Yoshida S, Akiyama K, Arite T, et al (2008) Inhibition of shoot branching by new terpenoid plant hormones. Nature 455: 195–200. 18690207 10.1038/nature07272

[11] Rasmussen A, Mason MG, Brewer PB, Herold S, Agusti J, et al (2012) Strigolactones suppress adventitious rooting in *Arabidopsis* and pea. Plant Physiol 158: 1976–1987. 22323776 10.1104/pp.111.187104 PMC3320200

[12] Mayzlish-Gati E, De Cuyper C, Goormachtig S, Beeckman T, Vuylsteke M, et al (2012) Strigolactones Are Involved in Root Response to Low Phosphate Conditions in Arabidopsis. Plant Physiol 160: 1329–1341. 22968830 10.1104/pp.112.202358 PMC3490576

[13] Arite T, Umehara M, Ishikawa S, Hanada A, Maekawa M, et al (2009) D14, a Strigolactone-Insensitive Mutant of Rice, Shows an Accelerated Outgrowth of Tillers. Plant Cell Physiol 50: 1416–1424. 19542179 10.1093/pcp/pcp091

[14] Waters MT, Nelson DC, Scaffidi A, Flematti GR, Sun YKM, et al (2012) Specialisation within the DWARF14 protein family confers distinct responses to karrikins and strigolactones in Arabidopsis. Development 139: 1285–1295. 22357928 10.1242/dev.074567

[15] Hamiaux C, Drummond RS, Janssen BJ, Ledger SE, Cooney JM, et al (2012) DAD2 is an alpha/beta hydrolase likely to be involved in the perception of the plant branching hormone, strigolactone. Curr Biol 22: 2032–2036. 22959345 10.1016/j.cub.2012.08.007

[16] Scaffidi A, Waters MT, Bond CS, Dixon KW, Smith SM, et al (2012) Exploring the molecular mechanism of karrikins and strigolactones. Bioorg Med Chem Lett 22: 3743–3746. 22542018 10.1016/j.bmcl.2012.04.016

[17] Waters MT, Scaffidi A, Flematti GR, Smith SM (2012) Karrikins force a rethink of strigolactone mode of action. Plant Signal Behav 7: 969–972. 22827937 10.4161/psb.20977 PMC3474697

[18] Goddard-Borger ED, Ghisalberti EL, Stick RV (2007) Synthesis of the germination stimulant 3-methyl-2H-furo[2,3-c]pyran-2-one and analogous compounds from carbohydrates. Eur J Org Chem 2007: 3925–3934.

[19] Gill SC, von Hippel PH (1989) Calculation of protein extinction coefficients from amino acid sequence data. Anal Biochem 182: 319–326. 2610349 10.1016/0003-2697(89)90602-7

[20] Jancarik J, Pufan R, Hong C, Kim SH, Kim R (2004) Optimum solubility (OS) screening: an efficient method to optimize buffer conditions for homogeneity and crystallization of proteins. Acta Crystallogr D 60: 1670–1673. 15333951 10.1107/S0907444904010972

[21] Kabsch W (2010) XDS. Acta Crystallographica D 66: 125–132. 10.1107/S0907444909047337 PMC2815665 20124692

[22] Evans P (2006) Scaling and assessment of data quality. Acta Crystallogr D 62: 72–82. 16369096 10.1107/S0907444905036693

[23] Winn MD, Ballard CC, Cowtan KD, Dodson EJ, Emsley P, et al (2011) Overview of the CCP4 suite and current developments. Acta Crystallogr D 67: 235–242. 21460441 10.1107/S0907444910045749 PMC3069738

[24] Murshudov GN, Vagin AA, Dodson EJ (1997) Refinement of macromolecular structures by the maximum-likelihood method. Acta Crystallogr D 53: 240–255. 15299926 10.1107/S0907444996012255

[25] Kaneko T, Tanaka N, Kumasaka T (2005) Crystal structures of RsbQ, a stress-response regulator in *Bacillus subtilis* . Protein Sci 14: 558–565. 15632289 10.1110/ps.041170005 PMC2253412

[26] Emsley P, Cowtan K (2004) Coot: model-building tools for molecular graphics. Acta Crystallogr D 60: 2126–2132. 15572765 10.1107/S0907444904019158

[27] Vagin AA, Steiner RA, Lebedev AA, Potterton L, McNicholas S, et al (2004) REFMAC5 dictionary: organization of prior chemical knowledge and guidelines for its use. Acta Crystallogr D 60: 2184–2195. 15572771 10.1107/S0907444904023510

[28] Bricogne G, Blanc E, Brandl M, Flensburg C, Keller P, et al.. (2011) BUSTER version 1.10.0. Cambridge, United Kingdom: Global Phasing Ltd.

[29] Kleywegt GJ (1996) Use of non-crystallographic symmetry in protein structure refinement. Acta Crystallogr D 52: 842–857. 15299650 10.1107/S0907444995016477

[30] Kleywegt GJ, Jones TA (1994) Detection, Delineation, Measurement and Display of Cavities in Macromolecular Structures. Acta Crystallogr D 50: 178–185. 15299456 10.1107/S0907444993011333

[31] Schrodinger The PyMOL Molecular Graphics System, Version 1.2r3pre. LLC.

[32] Chen VB, Arendall WB, Headd JJ, Keedy DA, Immormino RM, et al (2010) MolProbity: all-atom structure validation for macromolecular crystallography. Acta Crystallogr D 66: 12–21. 20057044 10.1107/S0907444909042073 PMC2803126

[33] Bond CS, Schuttelkopf AW (2009) ALINE: a WYSIWYG protein-sequence alignment editor for publication-quality alignments. Acta Crystallogr D 65: 510–512. 19390156 10.1107/S0907444909007835

[34] Nardini M, Dijkstra BW (1999) Alpha/beta hydrolase fold enzymes: the family keeps growing. Curr Opin Struct Biol 9: 732–737. 10607665 10.1016/s0959-440x(99)00037-8

[35] Cummins I, McAuley K, Fordham-Skelton A, Schwoerer R, Steel PG, et al (2006) Unique regulation of the active site of the serine esterase S-formylglutathione hydrolase. J Mol Biol 359: 422–432. 16626737 10.1016/j.jmb.2006.03.048

[36] Peng Q, Zhang X, Shang M, Wang X, Wang G, et al (2011) A novel esterase gene cloned from a metagenomic library from neritic sediments of the South China Sea. Microb Cell Fact 10: 95. 22067554 10.1186/1475-2859-10-95 PMC3226443

[37] Derewenda ZS, Derewenda U, Kobos PM (1994) (His)C epsilon-H...O = C < hydrogen bond in the active sites of serine hydrolases. J Mol Biol 241: 83–93. 8051710 10.1006/jmbi.1994.1475

[38] Ash EL, Sudmeier JL, Day RM, Vincent M, Torchilin EV, et al (2000) Unusual 1H NMR chemical shifts support (His) C(epsilon) 1...O = = C H-bond: proposal for reaction-driven ring flip mechanism in serine protease catalysis. Proc Natl Acad Sci U S A 97: 10371–10376. 10984533 10.1073/pnas.97.19.10371 PMC27031

[39] Nelson DC, Scaffidi A, Dun EA, Waters MT, Flematti GR, et al (2011) F-box protein MAX2 has dual roles in karrikin and strigolactone signaling in Arabidopsis thaliana. Proc Natl Acad Sci U S A 108: 8897–8902. 21555559 10.1073/pnas.1100987108 PMC3102411

